# Risk factors when considering heart transplants with donors aged 45 or above: Development of a Novel Mortality Risk Score using UNOS data

**DOI:** 10.1016/j.jhlto.2026.100497

**Published:** 2026-01-23

**Authors:** Anh Nguyen, Abbas Rana, Alexis Shafii, Gabriel Loor, Andrew Civitello, Todd Rosengart, Kenneth Liao

**Affiliations:** a Division of Cardiothoracic Transplantation and Circulatory Support, Department of Surgery, Baylor College of Medicine, Houston, Texas; b Division of Abdominal Transplantation, Department of Surgery, Baylor College of Medicine, Houston, Texas; c Division of Cardiology, Department of Medicine, Baylor College of Medicine, Houston, Texas; d Division of Cardiothoracic Surgery, Department of Surgery, Baylor College of Medicine, Houston, Texas

**Keywords:** heart transplant, survival, mortality, risk score, donor age

## Abstract

**Background:**

Current International Society for Heart and Lung Transplantation guidelines support the use of donor hearts aged ≥45 if significant coronary artery disease is excluded and ischemic time remains <4 hours. This study aimed to identify additional risk factors affecting patient survival and develop a risk score for recipients of older donor hearts.

**Methods:**

We performed a retrospective cohort study of adult heart transplants using donors aged ≥45 from January 2000 to June 2024, using United Network for Organ Sharing data. Multivariable Cox regression identified risk factors for mortality, and the most significant variables were used to construct a risk score.

**Results:**

Of 58,859 adult heart transplants, 9,843 (16.7%) involved donors aged ≥45. Recipient median age was 58 years (interquartile range, 51-64). One-, 5-, and 10-year patient survival rates were 87.0%, 73.3%, and 53.5%, respectively. Statistically significant risk factors included ischemic time ≥4 hours (HR = 1.11), cytomegalovirus (CMV)-positive donor status (hazard ratio [HR] = 1.11), donor cigarette use (HR = 1.10) and recipient factors: age >55 (HR = 1.37), Black race (HR = 1.19), obesity (HR = 1.20), diabetes (HR = 1.27), pretransplant dialysis (HR = 1.44), pretransplant mechanical ventilator use (HR = 1.37), and prior cardiac surgery (HR = 1.28). Model discrimination was moderate with C-statistics of 0.58.

**Conclusions:**

When considering heart transplants with donors aged ≥45, we should screen for ischemic time ≥4 hours, CMV-positive donor, donor cigarette use, and the following recipient factors: age >55, Black race, obesity, diabetes, pretransplant dialysis or mechanical ventilation, and prior cardiac surgery.

## Background

Heart failure continues to pose a significant global health challenge, affecting more than 64 million individuals worldwide and over 6.7 million adults annually in the United States.^1^ Heart transplantation remains the most effective therapy for patients with advanced heart failure, offering marked improvements in both survival and quality of life. However, the demand for donor hearts consistently surpasses the available supply.^2^ Alarmingly, more than 30% of patients on the transplant waiting list face mortality within 1 year if a suitable donor heart is not found.^3^ The use of extended donor criteria has included older donors in efforts to expand the donor pool. While some studies reported that older heart donors were associated with worse post-transplant survival,^4^, ^5^, ^6^, ^7^, ^8^, ^9^, ^10^ others showed that older donor hearts may achieve comparable perioperative outcomes and survival with careful selection.^11^, ^12^ The current International Society for Heart and Lung Transplantation (ISHLT) guidelines for donor heart selection stated that there was no established upper age limit and donors ≥45 years could be used after screening for significant coronary artery disease and short ischemic times could be expected.^13^ Our study aims to assess additional risk factors that may influence heart transplant patient survival with older donors and develop a corresponding mortality risk score to assist clinicians while considering heart donors aged ≥45 years.

## Methods

### Data source

It was a retrospective cohort study using data from the United Network for Organ Sharing Standard Transplant Analysis and Research dataset. We evaluated adult heart transplants with donors aged ≥45 years between January 2000 and June 2024. Donor information obtained from the database included age, gender, weight, body mass index, mechanism of death, hypertension, coronary artery disease, cigarette use, left ventricular ejection fraction, cytomegalovirus (CMV) status, and ischemic time. Recipient information obtained from the database included age, gender, ethnicity, weight, body mass index, diabetes, history of cardiac surgery, pretransplant dialysis, inotropic support, mechanical ventilation, extracorporeal membrane oxygenation (ECMO), or ventricular assist device (VAD), end listing status, and mortality. Transplant year was grouped into 5 transplant eras: 2000-2005, 2006-2010, 2011-2015, 2016-2020, and 2021-2024.

### Statistical analysis

Donor and recipient characteristics were reported as frequencies and proportions for categorical variables and as median and interquartile range (IQR) for continuous variables. Multivariable Cox proportional hazards regression was used to identify risk factors associated with mortality. The model was adjusted for ischemic time, CMV mismatch, gender mismatch, donor/recipient weight ratio, donor hypertension, donor left ventricular ejection fraction, donor diabetes, donor cigarette and cocaine use, donor cause of death, recipient age, recipient gender, recipient race, recipient obesity, recipient pretransplant dialysis/cardiac surgery/mechanical ventilation/ECMO/VAD/IV inotropes, recipient end listing status, and transplant era. Statistically significant and clinically relevant variables were kept optimizing the model performance with C-statistics of 0.58. To develop a clinically friendly risk score, we ran a Cox regression model with the significant variables in addition to recipient ECMO and converted their beta-coefficients in the model to integer risk scores according to a well-established methodology.^14^ The total risk score of each patient was calculated based on these 9 risk factors and then classified as low (<25% percentile), moderate (25%-75% percentile), and high risk (>75% percentile). Kaplan-Meier graphs were used to present patient survival by these 3 risk categories and the log-rank test to compare overall survival. All tests were 2-tailed with an alpha level of 0.05. Analyses were performed on Stata version 18.0 (Stata Corp LLC, College Station, TX).

This study was approved with a waiver of patient consent and Health Insurance Portability and Accountability Act authorization by the Institutional Review Board for Human Subject Research for Baylor College of Medicine and Affiliated Hospitals (H-51123, approved on November 29, 2022).

## Results

### Donor and recipient characteristics

Of the 58,859 adult heart transplants performed during the study period, 9,843 (16.7%) involved donors aged 45 years or older. The median donor age was 49 years (IQR, 47-53). Gender mismatch between donor and recipient occurred in 27.8% of cases, and 11.9% of donors had a weight less than 80% of the recipients. A history of hypertension was present in 35.5% of donors. Most donors had preserved left ventricular function, with 99.1% exhibiting an ejection fraction >50%. Coronary artery stenosis >50% was observed in 9.8% of donors. Additionally, 6.8% of donors had a history of diabetes. Substance use was notable, with 32.3% reporting cigarette use and 17.5% reporting cocaine use. The most common causes of donor death were stroke (47.1%), head trauma (29.6%), and anoxia (21.0%), with other causes accounting for 2.3%. The median ischemic time was 3.3 hours (IQR, 2.5-3.9) and 2,499 transplants (25.4%) had ischemic time ≥4 hours. CMV serostatus combinations included donor(−)/recipient(−) in 16.0%, donor(−)/recipient(+) in 21.0%, donor(+)/recipient(+) in 38.9%, and donor(+)/recipient(−) in 24.1% of cases.

Recipients had a median age of 58 years (IQR, 51-64), and 26.7% were female. The majority were White (68.6%), followed by Black (19.5%), Hispanic (7.9%), Asian (3.0%), American Indian (0.2%), Pacific Islander (0.4%), and Multiracial (0.3%). Comorbid conditions included obesity in 29.1%, diabetes in 28.6%, and pretransplant dialysis in 4.8%. A history of pretransplant cardiac surgery was present in 39.6% of recipients, while 40.5% required inotropic support, 25.8% had a VAD, 2.3% were on mechanical ventilation, and 1.8% received ECMO. At the time of listing, 62.0% were status 1, followed by status 2 (22.9%), status 3 (5.7%), status 4 (6.8%), status 5 (0.4%), and status 6 (2.1%). Detailed recipient characteristics are presented in Table 1.

**Table 1:** Baseline Characteristics of Donors Aged ≥45 Years and Their Heart Recipients

Characteristic	Summary Statistic
*Donors*	
Age (years), median (IQR)	49 (47-53)
Gender mismatch, N (%)	2,736 (27.8)
Donor/recipient weight <80%, N (%)	1,167 (11.9)
Hypertension, N (%)	3,451 (35.5)
Left ventricular ejection fraction >50%	9,614 (99.1)
Coronary artery stenosis >50%, N (%)	65 (9.8)
Ischemic time (hours), median (IQR)	3.3 (2.5-3.9)
CMV status, N (%)	
Donor (−)/recipient (−)	1,471 (16.0)
Donor (−)/recipient (+)	1,940 (21.0)
Donor (+)/recipient (+)	3,582 (38.9)
Donor (+)/recipient (−)	2,223 (24.1)
Diabetes	665 (6.8)
Cigarette use	3,105 (32.3)
Cocaine use	1,588 (17.5)
Cause of death	
Anoxia	2,061 (21.0)
Stroke	4,637 (47.1)
Head trauma	2,908 (29.6)
Others	232 (2.3)

*Recipients*	
Age (years), median (IQR)	58 (51-64)
Female, N (%)	2,625 (26.7)
Ethnicity, N (%)	
White	6,756 (68.6)
Black	1,914 (19.5)
Hispanic	778 (7.9)
Asian	298 (3.0)
American Indian	25 (0.2)
Pacific Islander	40 (0.4)
Multiracial	30 (0.3)
Obesity, N (%)	2,863 (29.1)
Diabetes, N (%)	2,796 (28.6)
Pretransplant dialysis, N (%)	461 (4.8)
Pretransplant cardiac surgery, N (%)	3,151 (39.6)
Pretransplant mechanical ventilator, N (%)	229 (2.3)
Pretransplant ECMO, N (%)	117 (1.8)
Pretransplant inotropic support, N (%)	3,989 (40.5)
Pretransplant ventricular assist device, N (%)	2,538 (25.8)
End listing status, N (%)	
1	6,105 (62.0)
2	2,259 (22.9)
3	562 (5.7)
4	667 (6.8)
5	39 (0.4)
6	209 (2.1)
Transplant era	
2000-2005	2,162 (22.0)
2006-2010	1,714 (17.4)
2011-2015	1,920 (19.5)
2016-2020	2,254 (22.9)
2021-2024	1,793 (18.2)

Abbreviations: CMV, cytomegalovirus; ECMO, extracorporeal membrane oxygenation; IQR, interquartile range.

### Mortality predictors of heart transplants with donors aged ≥45 years

One-, 5-, and 10-year survival rates were 87.0% (95% confidence interval [CI] 86.3-87.7), 73.3% (95% CI 72.4-74.3), and 53.5% (95% CI 52.2-54.7), respectively.

Donor coronary artery stenosis >50% was associated with a nonstatistically significant risk of post-transplant mortality (hazard ratio [HR] = 1.35, 95% CI 0.98-1.86, *p* = 0.06). Due to the limited availability of angiographic data present in only 662 donors (6.7%), this variable was not included in the multivariable analysis.

In the multivariable Cox proportional hazards model evaluating heart transplant outcomes with donors aged ≥45 years, several donor and recipient factors were significantly associated with post-transplant survival. Among donor characteristics, ischemic time ≥4 hours was associated with increased mortality risk (HR = 1.12; 95% CI, 1.03-1.23; *p* = 0.011). CMV serostatus mismatch also influenced outcomes: compared to donor(−)/recipient(−) pairs, donor(+)/recipient(+) combinations were associated with higher mortality (HR = 1.8; 95% CI, 1.05-1.32; *p* = 0.007), as were donor(+)/recipient(−) pairs (HR = 1.14; 95% CI, 1.00-1.29; *p* = 0.044). Additionally, donor cigarette use was associated with a 10% increase in mortality risk (HR = 1.10; 95% CI, 1.01-1.19; *p* = 0.028).

Recipient characteristics showed stronger associations with post-transplant outcomes. Increasing age was a significant predictor of mortality, with recipients aged 56 to 65 (HR = 1.35; 95% CI, 1.19-1.53; *p* = < 0.001) and >65 years (HR = 1.61; 95% CI, 1.41-1.85; *p* < 0.001) having elevated risk compared to those ≤35 years. Black race (HR = 1.1; 95% CI, 1.10-1.4; *p* < 0.001), obesity (HR = 1.21; 95% CI, 1.1-1.32; *p* < 0.001), and diabetes (HR = 1.26; 95% CI, 1.17-1.38; *p* < 0.001) were also independently associated with worse survival. Pretransplant dialysis (HR = 1.46; 95% CI, 1.25-1.71; *p* < 0.001), cardiac surgery (HR = 1.26; 95% CI, 1.17-1.37; *p* < 0.001), and mechanical ventilator support (HR = 1.45; 95% CI, 1.09-1.93; *p* < 0.011) were significant risk factors. Other donor and recipient factors, including gender mismatch, donor hypertension, donor diabetes, donor cocaine use, donor cause of death, and use of inotropes, VADs, or ECMO, were not significantly associated with survival. The final model demonstrated moderate discriminatory ability, with a C-statistic of 0.58 (95% CI 0.57-0.60). Full details are presented in Table 2.

**Table 2:** Multivariable Survival Analysis for Heart Transplants With Donors Aged ≥45 Years

Risk factors	Hazard ratio [95% CI]	*p*-value
*Donors*		
Ischemic time ≥4 hours	1.12 [1.03-1.23]	0.011
CMV		
Donor (−)/recipient (−)	Ref	
Donor (−)/recipient (+)	1.07 [0.94-1.22]	0.313
Donor (+)/recipient (+)	1.18 [1.05-1.33]	0.007
Donor (+)/recipient (−)	1.14 [1.00-1.29]	0.044
Gender mismatch	0.99 [0.91-1.08]	0.835
Donor/recipient weight ratio <0.8	1.02 [0.91-1.16]	0.700
Donor hypertension	1.03 [0.95-1.12]	0.476
Left ventricular ejection fraction >50%	1.06 [0.69-1.63]	0.795
Diabetes	1.01 [0.87-1.17]	0.861
Cigarette use	1.10 [1.01-1.19]	0.028
Cocaine use	0.92 [0.83-1.03]	0.146
Cause of death		
Anoxia	Ref	
Stroke	1.01 [0.91-1.13]	0.801
Head trauma	1.00 [0.89-1.12]	0.978
Others	1.04 [0.80-1.36]	0.761

*Recipients*		
Age		
≤45	Ref	
46-55	1.07 [0.94-1.23]	0.275
56-65	1.35 [1.19-1.53]	<0.001
>65	1.61 [1.41-1.85]	<0.001
Female	0.94 [0.86-1.03]	0.169
Black	1.21 [1.10-1.34]	<0.001
Obesity	1.21 [1.11-1.32]	<0.001
Diabetes	1.26 [1.17-1.38]	<0.001
Pretransplant dialysis	1.46 [1.25-1.71]	<0.001
Pretransplant cardiac surgery	1.26 [1.17-1.37]	<0.001
Pretransplant mechanical ventilator	1.45 [1.09-1.93]	0.011
Pretransplant ECMO	1.26 [0.89-1.79]	0.186
IV inotropes at transplant	0.95 [0.87-1.03]	0.212
VAD	0.99 [0.89-1.10]	0.887
End status		
1	0.94 [0.79-1.12]	0.517
2	0.98 [0.82-1.18]	0.833
3-6	Ref	
Transplant era		
2000-2005	Ref	
2006-2010	0.99 [0.86-1.15]	0.917
2011-2015	0.96 [0.82-1.13]	0.627
2016-2020	0.91 [0.77-1.10]	0.313
2021-2024	0.82 [0.64-1.04]	0.104

Abbreviations: CMV, cytomegalovirus; ECMO, extracorporeal membrane oxygenation; IV, intravenous; VAD, ventricular assist device.

### Mortality risk score for adult heart transplant with donors aged ≥45 years

**Figure 1:**
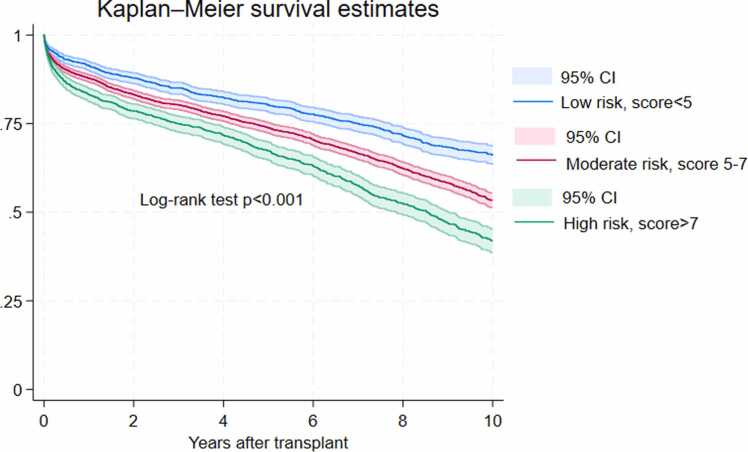
Heart transplant survival with donors aged ≥45 years by risk categories. Patients were grouped into low risk (<5), moderate risk (5-7), and high risk (>7) based on cumulative risk scores. The high-risk group demonstrated significantly reduced survival compared to low- and moderate-risk groups (*p* < 0.001), supporting the prognostic utility of the risk score.

Pretransplant ECMO was not a statistically significant risk factor (*p* = 0.183), probably due to the fact that only 117 patients (1.8%) were on ECMO. However, given its clinical relevance and relatively large effective size (HR = 1.25; 95% CI, 0.90-1.73), we still included it together with 10 other statistically significant variables in the multivariable model to develop the risk score. Three donor-related factors (ischemic time ≥4 hours, CMV-positive serostatus, and cigarette use) were each assigned a score of 1 point. Recipient-related factors contributed more substantially to overall risk. Recipient diabetes, pretransplant ECMO, and history of cardiac surgery were each assigned 3 points, while Black race and obesity contributed 2 points each. The highest individual scores were attributed to age >55, pretransplant dialysis, and mechanical ventilator support, each contributing 4 points. Based on cumulative scores, patients were stratified into 3 risk categories: low risk (total score <5), moderate risk (score 5-7), and high risk (score >7). Details are presented in Table 3. Kaplan-Meier analysis and log-rank test revealed significantly reduced survival in the high-risk group (*p* < 0.001), validating the utility of the score for clinical risk stratification (Figure 1). One-, 5, and 10-year survival rates by risk categories were presented in Table 4.

**Table 3:** Multivariable Model for the 11 Most Significant Factors and Their Corresponding Risk Scores

Risk factors	Hazard ratio [95% CI]	*p*-value	Coefficients	Score
*Donors*				
Ischemic time ≥4 hours	1.11 [1.02-1.21]	0.018	0.103	1
Donor CMV positive	1.11 [1.03-1.19]	0.009	0.100	1
Cigarette use	1.09 [1.01-1.18]	0.026	0.088	1

*Recipients*				
Age >55	1.37 [1.27-1.49]	<0.001	0.317	4
Black	1.19 [1.08-1.30]	<0.001	0.173	2
Obesity	1.20 [1.11-1.30]	<0.001	0.181	2
Diabetes	1.27 [1.17-1.37]	<0.001	0.238	3
Pretransplant dialysis	1.44 [1.24-1.69]	<0.001	0.368	4
Pretransplant cardiac surgery	1.29 [1.19-1.38]	<0.001	0.252	3
Pretransplant mechanical ventilator	1.37 [1.10-1.74]	0.011	0.313	4
Pretransplant ECMO	1.26 [0.92-1.73]	0.148	0.234	3
Low risk: total score <5; Moderate risk: total score 5-7; High risk: total score >7				

Abbreviations: CMV, cytomegalovirus; ECMO, extracorporeal membrane oxygenation.

**Table 4:** Survival Rates of Heart Transplants With Donors Aged ≥45 Years by Risk Categories

	Patient survival estimates with 95% confidence intervals		
Time frame (years)	Low risk (risk score <5)	Moderate risk (risk score 5-7)	High risk (risk score >7)
1	91.5 (90.2-92.7)	88.0 (86.9-89.1)	82.9 (81.1-84.6)
5	80.0 (78.0-81.9)	79.0 (72.6-75.7)	67.4 (64.9-69.7)
10	64.7 (62.0-67.3)	54.4 (52.2-56.5)	42.6 (39.4-45.7)

## Discussion

In response to the persistent shortage of donor hearts, there has been growing interest in expanding the donor pool by utilizing older donor hearts. The most recent ISHLT guidelines do not impose an upper age limit for donor heart selection and recommend considering donors aged ≥45 years, provided they are appropriately screened for coronary artery disease and short ischemic times (<4 hours) are anticipated.^13^ In our cohort, the oldest donor was 73 years old, and a total of 9,843 heart transplants (16.7% of all adult transplants between January 2000 and June 2024) involved donors aged ≥45 years. These organs represent a valuable resource that might otherwise have been discarded due to age alone.

Historically, advanced donor age has been associated with increased morbidity and mortality following heart transplantation.^4^, ^5^, ^6^, ^7^, ^9^, ^10^ However, recent studies using propensity score matching have demonstrated that, with careful donor-recipient matching, recipients of older donor hearts can achieve survival outcomes comparable to those receiving younger hearts.^11^, ^12^ These findings support the notion that older donor hearts, when appropriately selected, can be safely used in suitable candidates.

We found 10 significant risk factors associated with increased mortality in heart transplants with donors aged ≥45 years: ischemic time ≥4 hours, CMV-positive donor, donor cigarette use and the following recipient factors: age>55, Black race, obesity, diabetes, pretransplant dialysis, mechanical ventilation support, and prior cardiac surgery. Though not statistically significant, pretransplant ECMO was included in our risk score model given its large effect size and clinical relevance. In total, there are 11 factors we recommend to screen when considering heart donors aged ≥45 years. To the best of our knowledge, our model has fewer variables than previously published risk scores while still maintains a similar C-statistics of 0.58 (95% CI 0.57-0.60).^15^, ^16^, ^17^, ^18^, ^19^, ^20^, ^21^, ^22^ This suggests our work may offer a more streamlined and clinically friendly tool.

While most of the risk factors in our model are consistent with those reported in existing literature, CMV-positive donor status stands out.^15^, ^16^, ^17^, ^18^, ^19^, ^20^, ^21^, ^22^ CMV-positive donor is a well-established risk factor for CMV disease, which might lead to graft rejection and cardiac allograft vasculopathy. However, recent studies showed no statistically significant effect of CMV-positive donor on heart transplant survival.^23^, ^24^, ^25^ It might be due to the protective effect of antiviral prophylaxis and treatment. Our findings on the significant role of CMV status on heart transplant mortality might be specific for older donors only. Prior research has shown that donor age is an independent predictor of reduced CAV-free survival, implying that older hearts may be more susceptible to CMV-related endothelial injury, a key mechanism in the development of CAV.^26^ Further studies are warranted to explore the interaction between donor age and CMV infection in heart transplant outcomes.

### Study limitations

This study is based on a retrospective analysis of the United Network for Organ Sharing registry data, which is subject to several inherent constraints. Missing data and inaccuracies in reporting may affect the reliability of certain variables, and incomplete follow-up can limit the assessment of long-term outcomes.

We believe fewer variables will make our risk score more clinically friendly and widely utilized. However, we might not have controlled other unmeasured confounders that might contribute to heart transplant survival. Our 10 recommended risk factors are by no means exhaustive. Each heart transplant team should consider other factors such as the availability of organ to a highly sensitized recipient and the survival chance of a patient while on the waiting list before deciding to accept or reject a heart donor ≥45 years. Despite a clinically important criterion to screen for in ISHLT guideline, due to the limited availability of angiographic data present in only 662 donors (6.7%), coronary artery stenosis was not included in the multivariable analysis and in the risk score.

## Conclusions

Building on the ISHLT recommendations of avoiding significant coronary artery disease (coronary artery stenosis >50%) and ischemic time ≥4 hours, we have developed a novel risk score for use when considering heart transplant with a donor heart >45 years old. This risk score added CMV-positive donor status, donor cigarette smoking, and the following recipient factors: age >55, Black race, obesity, diabetes, pretransplant dialysis, pretransplant mechanical ventilator support, pretransplant ECMO, and prior cardiac surgery. While further prospective validation is warranted, this clinically useful and simple risk score may be utilized when considering the use of older donors for heart transplant.

## CRediT authorship contribution statement

**Anh Nguyen:** Conceptualization, Methodology, Formal analysis, Data curation, Writing – original draft. **Abbas Rana:** Methodology, Resources, Writing – review & editing. **Alexis Shafii:** Methodology, Resources, Writing – review & editing. **Gabriel Loor:** Methodology, Resources, Writing – review & editing. **Andrew Civitello:** Methodology, Resources, Writing – review & editing. **Todd Rosengart:** Resources, Writing – review & editing, Supervision. **Kenneth Liao:** Conceptualization, Methodology, Resources, Writing – review & editing, Supervision.

## Financial disclosure statement

This project does not have funding support from any sources.

## Declaration of Competing Interests

The authors declare that they have no known competing financial interests or personal relationships that could have appeared to influence the work reported in this paper.
